# Effects of Konjac glucosylceramides on cognitive function in cognitively unimpaired older adults with subjective cognitive concerns: a randomized, double-blind, controlled pilot study

**DOI:** 10.1016/j.jnha.2026.100975

**Published:** 2026-09-09

**Authors:** Kohei Yuyama, Hui Sun, Koichi Eguchi, Kenichi Oe, Yuichi Ukawa, Mika Otsuki, Hiroyuki Nakai, Jinichi Isono, Takayasu Sekine, Toshifumi Imada, Akiko Tanaka, Hiroyo Kagami-Katsuyama, Naoyuki Honma, Jun Nishihara, Kenji Monde

**Affiliations:** a Graduate School of Life Science, Hokkaido University, Sapporo, Hokkaido, Japan; b Faculty of Advanced Life Science, Hokkaido University, 001-0021, Sapporo, Hokkaido, Japan; c Institute for the Promotion of Business-Regional Collaboration, Hokkaido University, Sapporo, Hokkaido, Japan; d Safety and Quality Assurance Headquarters, Quality Assurance Center Daicel Corporation, Himeji, Hyogo, Japan; e Healthcare SBU Business Strategy, R&D Daicel Corporation, Niigata, Japan; f Healthcare SBU Business Strategy, Business Planning Daicel Corporation, Tokyo, Japan; g Faculty of Health Sciences, Hokkaido University, Sapporo, Hokkaido, Japan; h D-LAB, Japan Tobacco Inc., 1-1, Toranomon 4-chome, Tokyo, Japan; i Food Technology Solution Department, Nishimoto Co., Ltd., Tokyo, Japan; j Health Information Science Center, Hokkaido Information University, Ebetsu, Hokkaido, Japan

**Keywords:** Glucosylceramide, Cognitive function, Amyloid biomarker, Randomized controlled trial, Preclinical Alzheimer’s disease

## Abstract

**Objectives:**

Diet-derived bioactive lipids may help maintain cognitive resilience during the preclinical stage of Alzheimer’s disease (AD). Konjac-derived glucosylceramide (kGlcCer), traditionally used for skin barrier support, has previously been shown to reduce brain amyloid accumulation in individuals with lower baseline amyloid burden. However, earlier studies used cognitive tests with limited sensitivity. The objective of this study was to investigate whether daily intake of kGlcCer improves cognitive performance and influences amyloid-related biomarkers in cognitively unimpaired older adults with subjective cognitive concerns.

**Design:**

A 24-week randomized, double-blind, placebo-controlled, parallel-group trial.

**Participants:**

Forty cognitively unimpaired older adults aged 60–80 years who reported subjective decline in memory or cognitive function were enrolled and randomly assigned to study groups.

**Intervention:**

Participants received placebo or kGlcCer at doses of 1.8, 3.6, or 5.4 mg/day for 24 weeks.

**Measurements:**

Cognitive outcomes were assessed using the Wechsler Memory Scale–Revised (WMS-R) Logical Memory I/II, Wechsler Adult Intelligence Scale–Fourth Edition (WAIS-IV) Coding, and Standard Verbal Paired-Associate Learning Test (S-PA). Brain amyloid accumulation was estimated using plasma amyloid-β (Aβ) composite biomarker levels.

**Results:**

In this exploratory pilot trial, kGlcCer intake was associated with changes in several cognitive measures, although consistent between-group superiority was not observed across all outcomes. The medium-dose group showed significantly greater improvement in WAIS-IV Coding performance compared with the placebo group. Although significant within-group improvements were observed in several measures in the high-dose group such as immediate and delayed verbal memory assessed by WMS-R Logical Memory I/II, as well as in unrelated word-pair learning on the S-PA, consistent placebo-adjusted between-group differences were not observed across these outcomes. Stratified analysis further revealed kGlcCer intake may be associated with favorable changes in amyloid-related biomarkers in high-dose participants with low baseline amyloid burden. No safety concerns related to kGlcCer intake were observed during the study period.

**Conclusion:**

These exploratory findings suggest that kGlcCer may be associated with changes in selected cognitive domains and amyloid-related biomarkers. However, the present pilot study was not powered to establish efficacy, and larger adequately powered randomized trials are required.

**Study ID:**

UMIN000051463, date of registration: 07-03-2023.

## 1 Introduction

Dementia comprises clinical syndromes marked by progressive decline in cognitive and functional abilities that interfere with independent living [1]. Among its causes, Alzheimer’s disease (AD) is the most prevalent, defined biologically by brain amyloid-β (Aβ) deposition and tau accumulation [2]. Longitudinal cohorts show that these lesions accrue years – often decades – before symptoms [3]. In this preclinical stage, individuals remain cognitively normal yet display biomarker evidence and subtle cognitive change detectable with sensitive tests [4,5]. This extended prodrome offers a window for interventions that can delay or blunt subsequent decline, particularly approaches that are safe, scalable, and compatible with long-term use.

Diet is a key, modifiable determinant of late-life cognition [6,7]. Patterns emphasizing bioactive-rich foods – polyphenols, carotenoids, vitamins, long-chain omega-3 fatty acids, and specific lipids – are associated with healthier brain aging [8]. Mechanistic studies suggest convergent actions on early AD biology: dampening neuroinflammation and oxidative stress; supporting mitochondrial function and synaptic plasticity; maintaining neurovascular integrity [9,10]. Diet– microbiome interactions may further shape immune signaling and blood–brain barrier function, linking peripheral metabolic status to central resilience. Within this context, functional food materials offer a pragmatic avenue for the preclinical phase of AD. Rather than single-target pharmacology, they provide multi-target, low-risk bioactives suitable for sustained, population-level use [11,12].

Ceramides are bioactive sphingolipids composed of a long-chain sphingoid base N-acylated with a fatty acid [13]. As central intermediates in sphingolipid metabolism, they sit at the hub of pathways leading to sphingomyelin and glycosphingolipids, and they influence membrane organization, signaling, apoptosis, and stress responses. Structural heterogeneity - chain length, saturation, and hydroxylation - confers distinct biophysical properties that shape lipid-raft dynamics and protein sorting [14]. In the epidermis, ceramides constitute the major lipid fraction of the stratum corneum and, together with cholesterol and free fatty acids, assemble into lamellar bilayers that limit transepidermal water loss and maintain barrier integrity [15]. Because of these biological roles, ceramides are widely used as functional food ingredients for skin hydration and barrier support [[16], [17], [18], [19]]. Products typically contain plant- or animal-derived ceramide species - most commonly glucosylceramides extracted from konjac, rice, corn, or citrus peels, and milk-derived ceramides [18,20,21]. As oral ingredients, ceramides are attractive because they are familiar, well tolerated, and amenable to long-term, population-level use [22].

Beyond dermatologic benefits, emerging work suggests potential neurocognitive relevance [23]. We and others have investigated konjac-derived glucosylceramide (kGlcCer) as a candidate functional component for brain health [24]. In AD model mice, short-term oral administration (1 mg/day for 2 weeks) reduced Aβ burden in the hippocampus and cerebral cortex and improved short-term memory compared with untreated controls. Mechanistically, one plausible pathway is enhancement of Aβ clearance via extracellular vesicles/exosomes in the brain parenchyma, although causal links require confirmation [25,26]. In human studies, previous exploratory findings suggested that oral kGlcCer was associated with reduced amyloid accumulation among individuals in the low-amyloid subgroup [27]. Together, these observations motivate evaluation of ceramide-based functional foods not only for skin barrier support but also as feasible, low-risk interventions targeting early processes relevant to cognitive health in older adults before objective cognitive impairment develops. Here, we conducted an exploratory pilot randomized controlled trial to investigate whether daily intake of kGlcCer is associated with changes in cognitive performance and amyloid-related biomarkers.

## 2 Materials and methods

### 2.1 Ethics statements

This study was approved by the Ethics Committee of Hokkaido Information University (approval no. 2023-02) and was registered in the University Hospital Medical Information Network Clinical Trials Registry (UMIN trial ID: UMIN000051463). The study was conducted in accordance with the Ethical Guidelines for Medical and Health Research Involving Human Subjects (the Japanese Ministry of Education, Culture, Sports, Science and Technology and the Ministry of Health, Labour and Welfare, February 28, 2017) and in compliance with the Declaration of Helsinki (the 64th WMA General Assembly, Fortaleza, October 2013).

### 2.2 Trial design

**Fig. 1:**
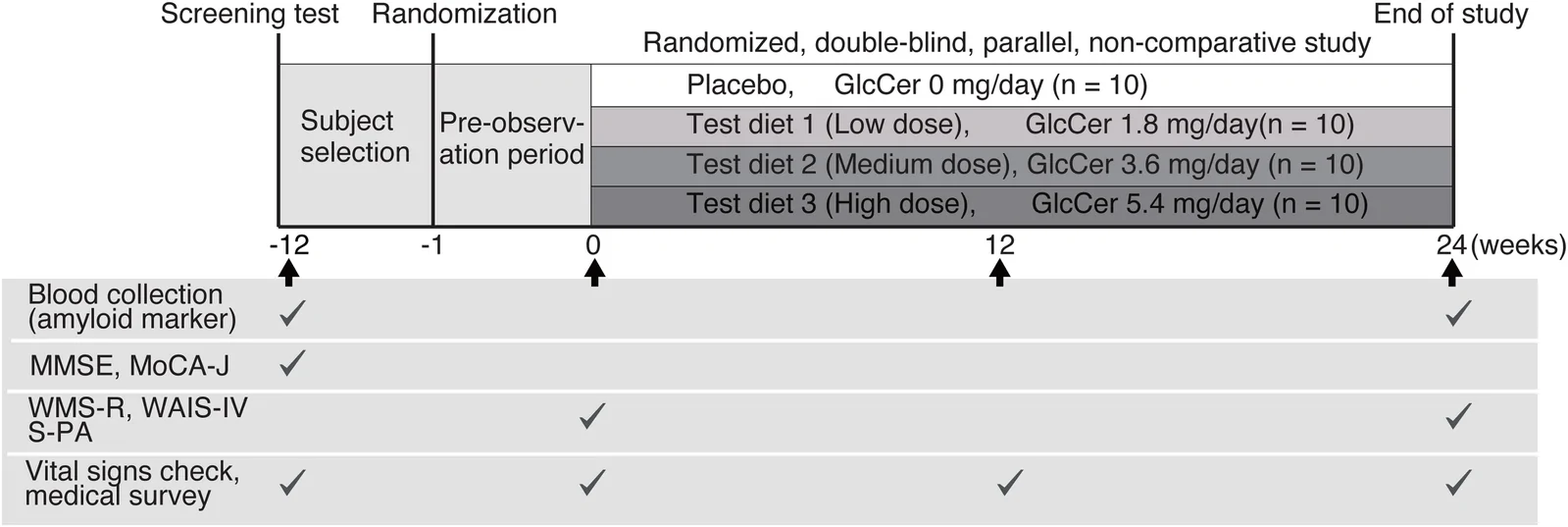
Schematic representation of the trial design.

This study was a 24-week, randomized, double-blind, parallel-group clinical trial. A schematic representation of the trial design is shown in Fig. 1.

### 2.3 Participants

Participants were Japanese men and women aged between 60 and 80 years who had subjective cognitive decline (SCD). They were individuals managed by the Health Information Science Research Center, Hokkaido Information University. Volunteers registered in the database were provided with the informed consent document approved by the Ethics Committee of Hokkaido Information University. The present trial was conducted in an independent cohort with no participant overlap with our previous study [27].

During the recruitment process, individuals who met the selection criteria listed in Supplementary Table S1 and did not meet any exclusion criteria, as confirmed by self-report, were invited for screening. Screening was conducted, and based on the results, 40 individuals who satisfied the inclusion criteria, met no exclusion criteria, and were deemed suitable for participation by the study physician were selected as study participants. In addition, during the study period (including the washout and intake phases), participants were instructed to follow the guidelines listed in Supplementary Table S2.

Participants were randomized using a stratified block randomization method, with sex, age, the Japanese version of the Montreal Cognitive Assessment (MoCA-J) [28] score and the composite amyloid-β biomarker as stratification factors. They were then divided into four groups: one that consumed a placebo and three that consumed the test food at doses of 1.8, 3.6, or 5.4 mg/day.

### 2.4 Test product

Konjac-derived glucosylceramide (kGlcCer, 100% pure) was supplied by Daicel Corporation (Tokyo, Japan). The test foods consisted of two types: an active test food and a placebo. The active test food was a jelly-type product containing kGlcCer, whereas the placebo was a jelly-type product without kGlcCer. The active product was formulated so that three sticks per day provided 1.8, 3.6, or 5.4 mg of kGlcCer. Both products were made to be packaged in stick-type aluminum laminate sachets (10 g per stick) and were made indistinguishable in appearance, shape, and flavor.

### 2.5 Assessment of cognitive functions

The subjects underwent a series of standardized neuropsychological assessments at two time points: at baseline prior to the initiation of the intervention and again at week 24 following completion of the test-food intake period. To assess key cognitive domains relevant to dementia while minimizing participant burden, a focused neuropsychological battery was used. Cognitive function was evaluated using tests targeting episodic memory, associative learning, and processing speed. Episodic memory impairment, a core feature of AD [29], was evaluated using the Logical Memory I and Logical Memory II subtests of the Wechsler Memory Scale–Revised (WMS-R) [30], measuring immediate and delayed recall of narrative information. In addition, processing speed and attention were evaluated using the Coding subtest of the Wechsler Adult Intelligence Scale–Fourth Edition (WAIS-IV) [31], a widely used measure of psychomotor speed, visual scanning, and sustained attention. Processing speed, which is known to be sensitive to cognitive aging [32], was assessed using the Coding subtest. Furthermore, associative memory and learning ability were examined using the Standard Verbal Paired-Associate Learning Test (S-PA) [33], which quantifies both related and unrelated paired-word learning. These tests capture complementary aspects of memory, including contextual memory and associative learning. These domains are among the key cognitive functions affected in dementia [34]; therefore, the present study focused on these domains.

### 2.6 Data quality assessment and handling of missing data

Cognitive test data were reviewed for documented procedural deficiencies that could compromise score reliability, including incomplete administration, deviation from standardized instructions, or interruption during testing. The same data-quality criteria were applied across all treatment groups. Exclusion decisions were made before statistical comparisons and while the principal investigator was blinded to treatment allocation. No missing or excluded values were imputed, and analyses were conducted using available evaluable data. The number of participants included in each analysis is reported in the corresponding tables.

### 2.7 Plasma Aβ biomarker

Plasma Aβ1‐40, Aβ1‐42, and amyloid precursor protein (APP)669‐711 levels were measured using immunoprecipitation-mass spectrometry (IP‐MS) based on matrix‐assisted laser desorption ionization time‐of‐flight MS (MALDI‐TOF MS). 1‐40/1‐42 and APP669‐711/Aβ1‐42 were calculated as the ratios of the normalized intensities of Aβ1‐40 and APP669‐711 to that of Aβ1‐42, respectively. The composite biomarker was computed by averaging the normalized scores of Aβ1‐40/1‐42 and APP669‐711/Aβ1‐42 [35].

### 2.8 Statistical analysis

All analyses were conducted at the Health Information Science Research Center, Hokkaido Information University. For the efficacy evaluation, between-group comparisons of the changes from baseline were performed using an independent two-sample t-test. Within-group comparisons between baseline and week 24 were conducted using a paired t-test for each group. For the safety evaluation, between-group comparisons of the changes from baseline were performed using an independent two-sample t-test. Within-group comparisons among the evaluation time points were conducted using a paired t-test for each group. As this study was designed as an exploratory pilot trial intended to generate hypotheses for future confirmatory studies, no adjustment for multiple comparisons was applied. Accordingly, all statistical findings should be interpreted as exploratory and hypothesis-generating rather than confirmatory evidence of efficacy. All statistical tests were performed using SPSS (version 25; IBM, Tokyo, Japan). All reported p-values are nominal because no adjustment for multiplicity was applied; nominal p-values < 0.05 were considered exploratory signals rather than confirmatory evidence.

## 3 Results

**Fig. 2:**
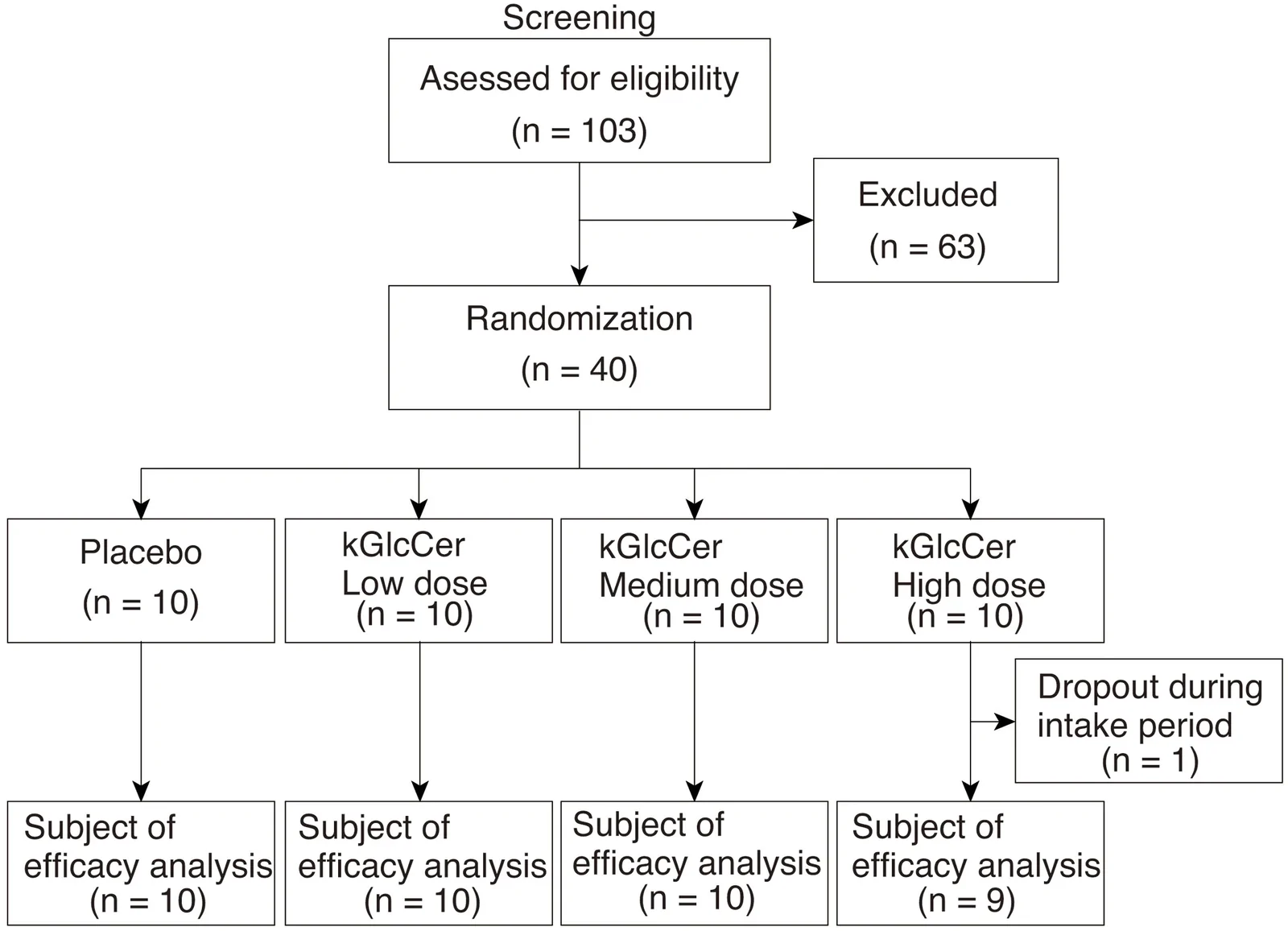
Schematic representation of the selection and distribution of the participants in the trial.

All 103 participants underwent the Mini-Mental State Examination (MMSE) [36] and MoCA-J. Also, the blood samples were assessed for the amyloid biomarker status. Forty individuals were selected based on the established selection and exclusion criteria and randomly assigned to four groups (n = 10 per group) by stratified permuted block randomization. Mean age, height, weight, body fat, body mass index (BMI), the values of the plasma Aβ composite biomarker, and MoCA-J score at baseline were not statistically different among four groups (Supplementary Table S3). No statistically significant differences were detected in the assessed baseline characteristics across the four groups. However, given the small sample size, the absence of statistical significance should not be interpreted as evidence of baseline equivalence, and descriptive differences were observed in several cognitive measures. During the study period, one participant withdrew, and a total of 39 participants (placebo group n = 10, low-dose group n = 10, medium-dose group n = 10, high-dose group n = 9) completed the experiment (Fig. 2). Sample sizes varied across cognitive outcomes because a small number of test results were excluded owing to documented procedural deficiencies during test administration. The same prespecified data-quality criteria were applied across all treatment groups, and exclusion decisions were made before statistical analysis while the principal investigator was blinded to treatment allocation. No values were imputed; analyses were based on available evaluable data, with the corresponding sample sizes reported in each table. No side effects were reported following administration of the kGlcCer in the participants throughout the study.

### 3.1 WMS-R

The Logical Memory I and II subtests of the Wechsler Memory Scale–Revised (WMS-R) assess different aspects of verbal episodic memory. Logical Memory I evaluates immediate recall and serves as an indicator of verbal memory. In contrast, Logical Memory II measures delayed recall and is used as an index of delayed memory ability. Together, these subtests provide important information about an individual’s capacity to encode and retain narrative information over time.

In Logical Memory I, no significant between-group differences were found at either time point for raw scores or percentile ranks (Table 1). Regarding within-group changes, a significant improvement was observed in the medium and high-dose group for the Logical Memory I raw score (p = 0.017 and p = 0.049). Within the high-dose group, percentile score of Logical Memory I also shows a significant change. Logical Memory II also showed no significant between-group differences at week 24. Within-group analysis revealed a significant increase in the high-dose group for the Logical Memory II raw score (p = 0.023) and percentile score (p = 0.024), whereas the other groups exhibited no significant changes.

**Table 1:** Assessment of cognitive changes by kGlcCer intake via WMS-R.

	Week	Group	Raw values			Change (0–24 week)			Between-group estimates (vs placebo)^1^					Within-group estimates (0 vs 24 week)^2^				
			N	Mean	SD	N	Mean	SD	Mean difference	95% CI Lower	95% CI Upper	Cohen's d	p-value	Mean difference	95% CI Lower	95% CI Upper	Cohen's d	p-value
WMS-R Logical Memory I Raw Score	0	Placebo	10	20.50	6.52													
		Low Dose	10	18.00	3.97													
		Medium dose	9	20.33	5.39													
		High dose	9	19.11	5.25													
	24	Placebo	10	21.70	9.01	10	1.20	5.37						−1.20	−5.042	2.642	−0.223	0.498
		Low Dose	10	20.50	6.29	10	2.50	5.25	−1.30	−6.292	3.692	−0.245	0.591	−2.50	−6.259	1.259	−0.476	0.167
		Medium dose	9	24.60	5.50	9	4.56	4.56	−3.36	−8.207	1.496	−0.670	0.163	−4.56	−8.059	−1.052	−0.999	*0.017
		High dose	9	22.11	7.30	9	3.00	3.87	−1.80	−6.381	2.781	−0.381	0.419	−3.00	−5.977	−0.023	−0.775	*0.049
WMS-R Logical Memory I Percentile	0	Placebo	10	52.20	26.93													
		Low Dose	10	40.40	18.90													
		Medium dose	9	52.00	28.89													
		High dose	9	43.67	21.87													
	24	Placebo	10	55.50	34.79	10	3.30	25.39						−3.30	−21.463	14.863	−0.130	0.691
		Low Dose	10	51.70	28.28	10	11.30	20.85	−8.00	−29.827	13.827	−0.344	0.451	−11.30	−26.214	3.614	−0.542	0.121
		Medium dose	9	67.60	18.57	9	17.33	24.11	−14.03	−38.072	10.006	−0.566	0.235	−17.33	−35.869	1.203	−0.719	0.063
		High dose	9	57.00	27.03	9	13.33	14.65	−10.03	−30.422	10.355	−0.477	0.314	−13.33	−24.598	−2.069	−0.910	*0.026
WMS-R Logical Memory II Raw Score	0	Placebo	10	15.70	9.39													
		Low Dose	9	14.00	3.97													
		Medium dose	10	17.30	6.29													
		High dose	9	13.78	6.12													
	24	Placebo	10	19.60	11.32	10	3.90	5.90						−3.90	−8.118	0.318	−0.661	0.066
		Low Dose	9	18.22	6.24	8	3.00	3.93	0.90	−4.257	6.057	0.175	0.716	−3.00	−6.284	0.284	−0.764	0.068
		Medium dose	10	21.30	6.25	10	4.00	5.73	−0.10	−5.565	5.365	−0.017	0.970	−4.00	−8.102	0.102	−0.697	0.055
		High dose	9	19.78	7.41	9	6.00	6.44	−2.10	−8.071	3.871	−0.341	0.468	−6.00	−10.952	−1.048	−0.931	*0.023
WMS-R Logical Memory II Percentile	0	Placebo	10	51.40	36.63													
		Low Dose	9	42.44	18.70													
		Medium dose	10	57.90	29.38													
		High dose	9	42.33	25.04													
	24	Placebo	10	62.50	35.60	10	11.10	25.55						−11.10	−29.377	7.177	−0.434	0.203
		Low Dose	9	62.78	27.52	8	14.13	18.71	−3.03	−25.963	19.913	−0.133	0.783	−14.13	−29.768	1.518	−0.755	0.070
		Medium dose	10	73.50	16.25	10	15.60	28.34	−4.50	−29.852	20.852	−0.167	0.714	−15.60	−35.876	4.676	−0.550	0.116
		High dose	9	67.33	27.07	9	25.00	27.03	−13.90	−39.354	11.554	−0.529	0.265	−25.00	−45.779	−4.221	−0.925	*0.024

95% CI, 95% confidence interval; *p < 0.05.

1 Between-group mean difference = placebo change - dose-group change; independent two-sample t-test.

2 Within-group mean difference = week 0 - week 24; paired t-test.

### 3.2 WAIS-IV

The Coding subtest of the Wechsler Adult Intelligence Scale-Fourth Edition (WAIS-IV) requires individuals to copy symbols that correspond to specific numbers. This timed task is a core measure of processing speed. It evaluates how quickly and accurately a person can perceive, match, and transcribe visual-motor information.

For the WAIS-IV Coding test, no significant between-group differences were observed in scaled scores (Table 2). However, a significant between-group difference was detected in change in the Coding raw score, such that the medium-dose group showed a significantly higher than placebo (p = 0.038). Within-group comparisons indicated that the medium-dose group demonstrated a significant improvement in raw score (p = 0.005) and scaled score (p = 0.004), while no significant within-group changes were observed in the placebo, low-dose, or high-dose groups.

**Table 2:** Assessment of cognitive changes by kGlcCer intake via WAIS-IV.

	Week	Group	Raw values			Change (0–24 week)			Between-group estimates (vs placebo)^1^					Within-group estimates (0 vs 24 week)^2^				
			N	Mean	SD	N	Mean	SD	Mean difference	95% CI Lower	95% CI Upper	Cohen's d	p-value	Mean difference	95% CI Lower	95% CI Upper	Cohen's d	p-value
WAIS-IV Coding Raw Score	0	Placebo	10	81.10	11.72													
		Low Dose	10	74.50	17.67													
		Medium dose	10	72.60	8.96													
		High dose	9	80.00	3.12													
	24	Placebo	10	83.10	11.48	10	2.00	4.37						−2.00	−5.13	1.13	−0.46	0.182
		Low Dose	10	78.20	21.85	10	3.70	10.46	−1.70	−9.51	6.11	−0.21	0.644	−3.70	−11.18	3.78	−0.35	0.292
		Medium dose	10	80.10	10.63	10	7.50	6.42	−5.50	−10.66	−0.34	−1.00	*0.038	−7.50	−12.09	−2.91	−1.17	*0.005
		High dose	9	84.56	10.17	9	4.56	9.18	−2.56	−9.40	4.28	−0.36	0.441	−4.56	−11.61	2.50	−0.50	0.175
WAIS-IV Coding Scaled Score	0	Placebo	10	13.10	1.91													
		Low Dose	10	11.30	3.50													
		Medium dose	10	11.20	1.55													
		High dose	9	12.22	0.97													
	24	Placebo	10	13.90	1.60	10	0.80	1.14						−0.80	−1.61	0.01	−0.71	0.053
		Low Dose	10	12.00	4.16	10	0.70	2.00	0.10	−1.43	1.63	0.06	0.892	−0.70	−2.13	0.73	−0.35	0.298
		Medium dose	10	12.60	1.90	10	1.40	1.17	−0.60	−1.69	0.49	−0.52	0.260	−1.40	−2.24	−0.56	−1.19	*0.004
		High dose	9	12.89	1.76	9	0.67	1.73	0.13	−1.27	1.54	0.09	0.843	−0.67	−2.00	0.67	−0.39	0.282

95% CI, 95% confidence interval; *p < 0.05.

1 Between-group mean difference = placebo change - dose-group change; independent two-sample t-test.

2 Within-group mean difference = week 0 - week 24; paired t-test.

### 3.3 S-PA

The S-PA (Related and Unrelated Word-Pair Association Test) is an assessment of verbal memory. It consists of 10 semantically related word pairs and 10 semantically unrelated word pairs, allowing evaluation of memory performance under differing associative conditions. This test serves as an important indicator of an individual’s ability to learn and recall verbal associations.

For the S-PA related word-pair test, no significant between-group differences or within-group changes were found in any group (Table 3). For the unrelated word-pair total score, significant between-group difference in change was detected, such that the high-dose group showed a significantly higher score than low-dose group (p = 0.023). Within-group analysis revealed that the high-dose group showed a significant improvement (p = 0.032). For the percentile score of unrelated word pairs, a significant between-group difference in change was detected, such that the high-dose group showed a significantly higher score than the placebo group (p = 0.030) and the low-dose group (p = 0.026). In addition, the high-dose group demonstrated a significant within-group increase (p = 0.005).

**Table 3:** Assessment of cognitive changes by kGlcCer intake via S-PA.

	Week	Group	Raw values			Change (0–24 week)			Between-group estimates (vs placebo)^1^					Within-group estimates (0 vs 24 week)^2^				
			N	Mean	SD	N	Mean	SD	Mean difference	95% CI Lower	95% CI Upper	Cohen's d	p-value	Mean difference	95% CI Lower	95% CI Upper	Cohen's d	p-value
S-PA Related Word-Pairs Total score	0	Placebo	10	28.9	1.6													
		Low Dose	10	28.8	1.4													
		Medium dose	9	27.78	3.11													
		High dose	9	28.22	2.86													
	24	Placebo	10	28.7	1.42	10	−0.2	1.03						0.2	−0.54	0.94	0.19	0.555
		Low Dose	10	27.9	3.38	10	−0.9	2.64	0.7	−1.19	2.59	0.35	0.446	0.9	−0.99	2.79	0.34	0.31
		Medium dose	9	27.8	2.3	9	0.11	2.67	−0.31	−2.23	1.61	−0.16	0.736	−0.11	−2.16	1.94	−0.04	0.904
		High dose	9	28.78	2.28	9	0.56	1.33	−0.76	−1.9	0.39	−0.64	0.183	−0.56	−1.58	0.47	−0.42	0.247
S-PA Unrelated Word-Pairs Total score	0	Placebo	10	13.6	5.3													
		Low Dose	10	15.5	6.59													
		Medium dose	10	12.8	10.94													
		High dose	9	12.11	8.3													
	24	Placebo	10	12.3	10.97	10	−1.3	9.07						1.3	−5.19	7.79	0.14	0.661
		Low Dose	10	12.5	9.32	10	−3	6.43	1.7	−5.69	9.09	0.22	0.634	3	−1.6	7.6	0.47	0.174
		Medium dose	10	12.2	10.23	10	−0.6	6.33	−0.7	−8.05	6.65	−0.09	0.844	0.6	−3.93	5.13	0.1	0.771
		High dose	9	15.11	9.52	9	3	3.46	−4.3	−11.05	2.45	−0.61	0.19	−3	−5.66	−0.34	−0.87	*0.032
S-PA Unrelated Word-Pairs Percentile Rank	0	Placebo	10	63.3	17.17													
		Low Dose	10	66.4	19.79													
		Medium dose	10	52.9	38.02													
		High dose	9	51.22	27.25													
	24	Placebo	10	53.2	34.02	10	−10	30.36						10.1	−11.62	31.82	0.33	0.32
		Low Dose	10	58.9	36.99	10	−7.5	25.85	−2.6	−29.09	23.89	−0.09	0.839	7.5	−10.99	25.99	0.29	0.383
		Medium dose	10	55.7	33.46	10	2.8	25.73	−12.9	−39.34	13.54	−0.46	0.319	−2.8	−21.21	15.61	−0.11	0.739
		High dose	9	66.78	31.7	9	15.56	12.34	−25.66	−48.38	−2.93	−1.08	*0.030	−15.56	−25.04	−6.07	−1.26	*0.005

95% CI, 95% confidence interval; *p < 0.05.

1 Between-group mean difference = placebo change - dose-group change; independent two-sample t-test.

2 Within-group mean difference = week 0 - week 24; paired t-test.

Across the cognitive assessments, placebo-adjusted between-group differences were limited and did not show a consistent pattern across outcomes or dose groups. A significant between-group difference was observed for the change in WAIS-IV Coding raw score in the medium-dose group compared with placebo. Several additional within-group improvements were observed; however, because these were not consistently accompanied by significant placebo-adjusted between-group differences, they should be regarded as exploratory observations rather than evidence of an intervention effect.

### 3.4 Aβ biomarker measurements

The blood Aβ composite biomarker serves as an indicator of amyloid accumulation in the brain and is used in the assessment of early AD diagnosis [2,36]. In our previous study, conducted under conditions similar to the present high-dose intake, the overall analysis showed no significant differences in biomarker values compared to the placebo group. However, a subgroup analysis, which classified subjects based on baseline Aβ composite biomarker values as either below or above the reference level, revealed significantly lower biomarker values in the kGlcCer intake group [27]. In the present study, while the total analysis showed a decreasing trend in the measured values and changes in the group consuming the test food, no statistically significant difference was observed (Table 4). However, in a predefined exploratory stratified analysis based on baseline Aβ composite biomarker levels, the high-dose group showed a significantly greater reduction than the placebo group among participants with lower baseline amyloid burden (p = 0.049). Because this subgroup analysis involved a limited number of participants, these findings should be interpreted as exploratory and hypothesis-generating.

**Table 4:** Changes in the values of plasma Aβ composite biomarker by kGlcCer administration.

	Week	Group	Raw values			Change (0–24 week)			Between-group estimates (vs placebo)^1^					Within-group estimates (0 vs 24 week)^2^				
			N	Mean	SD	N	Mean	SD	Mean difference	95% CI Lower	95% CI Upper	Cohen's d	p-value	Mean difference	95% CI Lower	95% CI Upper	Cohen's d	p-value
Plasma Aβ composite biomarker	0	Placebo	10	0.30	0.45													
		Low Dose	10	0.34	0.39													
		Medium dose	10	0.32	0.43													
		High dose	9	0.35	0.60													
	24	Placebo	10	0.26	0.43	10	−0.04	0.40						0.04	−0.24	0.33	0.11	0.735
		Low Dose	10	0.27	0.74	10	−0.07	0.86	0.02	−0.63	0.67	0.03	0.944	0.07	−0.55	0.68	0.08	0.816
		Medium dose	10	0.51	0.93	10	0.19	1.13	−0.23	−1.03	0.56	−0.28	0.546	−0.19	−1.00	0.62	−0.17	0.609
		High dose	9	0.11	1.09	9	−0.25	0.74	0.20	−0.36	0.77	0.35	0.456	0.25	−0.32	0.81	0.34	0.342
Plasma Aβ composite biomarker < 0.376	0	Placebo	5	−0.04	0.30													
		Low Dose	5	0.05	0.27													
		Medium dose	5	−0.03	0.25													
		High dose	4	−0.15	0.37													
	24	Placebo	5	0.16	0.31	5	0.21	0.27						−0.21	−0.54	0.13	−0.77	0.161
		Low Dose	5	0.45	0.82	5	0.40	0.71	−0.19	−0.98	0.60	−0.35	0.593	−0.40	−1.28	0.49	−0.56	0.282
		Medium dose	5	0.88	1.22	5	0.92	1.22	−0.71	−1.99	0.57	−0.81	0.238	−0.92	−2.43	0.59	−0.75	0.167
		High dose	4	−0.63	0.41	4	−0.47	0.57	0.68	0.01	1.36	1.60	*0.049	0.47	−0.44	1.38	0.83	0.196
Plasma Aβ composite biomarker 0.376 +	0	Placebo	5	0.65	0.25													
		Low Dose	5	0.63	0.24													
		Medium dose	5	0.68	0.18													
		High dose	5	0.76	0.39													
	24	Placebo	5	0.35	0.56	5	−0.29	0.36						0.29	−0.15	0.74	0.83	0.138
		Low Dose	5	0.10	0.71	5	−0.53	0.80	0.23	−0.67	1.13	0.38	0.567	0.53	−0.46	1.52	0.66	0.212
		Medium dose	5	0.14	0.31	5	−0.54	0.24	0.24	−0.20	0.69	0.80	0.240	0.54	0.24	0.84	2.23	*0.008
		High dose	5	0.69	1.13	5	−0.07	0.87	−0.23	−1.19	0.74	−0.34	0.603	0.07	−1.01	1.14	0.08	0.869

95% CI, 95% confidence interval; *p < 0.05.

1 Between-group mean difference = placebo change - dose-group change; independent two-sample t-test.

2 Within-group mean difference = week 0 - week 24; paired t-test.

## 4 Discussion

In this randomized, double-blind, placebo-controlled study, we examined whether daily intake of kGlcCer influences cognitive performance in cognitively unimpaired older adults at elevated risk of decline. By employing a battery of high-sensitivity neuropsychological assessments including WMS-R Logical Memory, WAIS-IV Coding, and the S-PA test, this exploratory pilot randomized controlled trial generated preliminary observations suggesting that daily kGlcCer intake may be associated with changes in selected cognitive domains. Because of the limited sample size, these findings should be interpreted as hypothesis-generating rather than confirmatory evidence of efficacy. A similar pattern was observed in our previous study [27], which was conducted in an independent cohort. These suggest that kGlcCer may be associated with favorable changes in selected cognitive measures and amyloid-related biomarkers. However, because this was an exploratory pilot study, these observations should not be interpreted as evidence of disease modification or prevention, and confirmation in larger prospective trials is required.

In recent years, substantial efforts have been devoted to developing cognitive test batteries capable of detecting the subtle cognitive changes characteristic of preclinical AD [37]. The cognitive battery used in this study assessed domains that overlap with those represented in composite measures developed for preclinical AD, such as the Preclinical Alzheimer’s Cognitive Composite (PACC), including episodic memory, processing speed, and associative learning [5,38]. However, the present battery was not designed as a direct equivalent of PACC, and its sensitivity to subtle longitudinal cognitive change has not been formally validated. Therefore, comparisons with PACC should be interpreted cautiously.

In the present study, the Aβ composite biomarker showed no statistically significant differences in either mean or change values across all intervention groups. Nonetheless, the high-dose group exhibited a modest trend toward decreased biomarker levels following intake. Based on the mean and standard deviation of the change values, a power analysis (two-sample t-test, 80% power, α = 0.05) indicated that a sample size of 100 participants per group would be required to detect a significant difference between the placebo and high-dose groups. Importantly, stratified analysis revealed that, among individuals with baseline Aβ composite values below the established threshold (0.376), the high-dose group demonstrated significant reduction in the biomarker levels compared with placebo, was consistent with the pattern observed in our previous study [27]. Since individuals in this lower-baseline subgroup are presumed to have early-stage amyloid accumulation, this exploratory observation raises the possibility that kGlcCer may have greater effects in individuals with lower baseline amyloid burden, although this hypothesis requires confirmation in adequately powered prospective studies. Although studies in AD mouse models have demonstrated reductions in brain amyloid pathology following kGlcCer administration [24], the present clinical study was not designed to evaluate disease modification. Therefore, whether similar biological effects occur in humans remains unknown and should be investigated in future adequately powered biomarker studies. Furthermore, advancements in blood-based biomarkers that more precisely estimate brain amyloid burden, including plasma pTau217 [39,40], have shown greater diagnostic sensitivity than Aβ measures and may enhance the ability to detect the effects of kGlcCer intake in future trials.

GlcCer, a glycosphingolipid containing a single glucose linked to a ceramide backbone, is hydrolyzed by intestinal digestive enzymes into glucose, fatty acids, and sphingoid bases, which are subsequently absorbed by enterocytes [41]. A fraction of these sphingoid bases - including those originating from plants - is resynthesized into ceramide, GlcCer, and more complex sphingolipids such as sphingomyelin, enabling these metabolites to circulate throughout the body and exert biological functions in various tissues [42,43]. Recent studies have shown that dietary plant sphingolipids, including kGlcCer, confer several beneficial effects, such as enhancement of the skin barrier and anticancer activity [16,44]. Moreover, evidence from cell culture and mouse models indicates that kGlcCer-derived sphingolipid components can cross the blood–brain barrier (BBB) and accumulate in the brain [45], suggesting their potential to modulate neural processes. Ceramide itself is known to participate in diverse physiological mechanisms, including anti-inflammatory signaling and neurite outgrowth [46,47], highlighting the importance of further investigation into mechanistic pathways underlying cognitive improvement. Of particular interest, konjac-derived ceramide (kCer) has been shown to induce the production of neuron-derived exosomes, which possess amyloid-β–binding capacity and facilitate its clearance; consistent with this, increased levels of such exosomes have been observed in the brains of kGlcCer-administered mice [24]. Although our measurement of serum Aβ-binding exosomes by ELISA did not reveal changes across groups in the present study (Supplementary Table S4), future work employing more sensitive quantification methods for brain-derived exosomes will be essential to elucidate whether exosome-mediated pathways contribute to the observed associations between kGlcCer intake and changes in cognitive performance or amyloid-related biomarkers.

Finally, the safety evaluation conducted throughout the 24-week intervention revealed no adverse events attributable to the test food, nor were any clinically significant abnormalities observed in physical examinations or clinical laboratory tests. Comprehensive analyses of safety-related parameters - including body composition indices (body weight, body fat percentage, BMI), vital signs (blood pressure, pulse rate), hematological measures (WBC, RBC, Hb, Ht, Plt), liver function markers (AST, ALT, γ-GTP, ALP, LDH, FIB-4 index), renal function markers (BUN, CRE, UA), lipid profiles (TC, LDL-C, HDL-C, TG), and glucose-related measures (random glucose, HbA1c) - revealed that although several parameters showed statistically significant within-group or between-group differences, all values remained within the normal clinical range and were not considered clinically relevant. Based on these findings, the principal investigator concluded that daily intake of 5.4 mg of konjac-derived glucosylceramide for 24 weeks presents no safety concerns. The clinical significance of the observed cognitive changes remains uncertain. Although effect sizes and 95% confidence intervals are provided to characterize the magnitude and precision of the findings, this study did not assess everyday cognitive functioning or prespecify thresholds for clinically meaningful change. Therefore, whether these changes translate into meaningful real-world cognitive benefits cannot be determined from the present study. These preliminary findings support further investigation of kGlcCer in larger adequately powered randomized clinical trials.

This study has several important limitations. First, the sample size was small (approximately 10 participants per group), limiting statistical power to detect modest intervention effects. Second, this study was designed as an exploratory pilot trial, and no adjustment for multiple comparisons was applied; therefore, statistically significant findings should be interpreted cautiously as hypothesis-generating observations. Accordingly, some statistically significant findings may represent false-positive results arising from multiple testing rather than true intervention effects and therefore require confirmation in larger independent studies. Furthermore, several observations were derived primarily from within-group analyses rather than consistent placebo-adjusted between-group differences. Such findings should therefore be interpreted cautiously because they may reflect practice effects, regression to the mean, natural fluctuation, or random variation rather than true intervention effects. Third, subgroup analyses further reduced sample sizes and require confirmation in adequately powered independent cohorts. In addition, descriptive baseline imbalances were observed in several cognitive outcomes. In particular, the lower baseline WAIS-IV Coding score in the medium-dose group may have contributed to the greater change from baseline through regression to the mean. Therefore, the change-score findings should be interpreted cautiously. Finally, the subgroup analysis according to baseline amyloid biomarker levels was exploratory and involved small sample sizes. Therefore, these findings should be interpreted cautiously and require independent confirmation in larger prospective studies. Larger multicenter randomized trials will be necessary to establish the efficacy of kGlcCer for cognitive function and amyloid-related biomarkers. Future large-scale studies will be essential to more precisely determine the extent to which long-term intake influences cognitive trajectories and brain amyloid accumulation.

## Author contributions

Conceptualization, K.Y., K.E., K.O., Y. U., H.N., J.I., T.S. and T.I.; methodology, M.O., A.T., H.K., N.H. and J.N.; formal analysis, H.K.; investigation, A.T.; principal investigation, J.N.; writing—original draft, K.Y.; writing—review and editing, H.S., K.E., K.O., Y.U., T.S., T.I., M.O. and A.T.; visualization, K.Y.; supervision, K.Y., Y. U., N.H. and K.M.; All authors have read and agreed to the published version of the manuscript.

## Informed consent statement

Informed consent was obtained from all subjects involved in the study.

## Declaration of Generative AI and AI-assisted technologies in the writing process

During the preparation of this work the authors used ChatGPT (AI language model) in order to help with grammar, spelling, and sentence structure. No analytical or interpretative content was produced by the model. After using this model, the authors reviewed and edited the content as needed and take full responsibility for the content of the publication.

## Funding

Daicel Corporation and Japan Tobacco Inc. provided research funding.

## Data statement

The datasets generated and/or analyzed during the current study are available from the corresponding author upon reasonable request.

## Declaration of competing interest

The authors declare no conflict of interest. Daicel Corporation and Japan Tobacco Inc. provided research funding but had no role in the study design, data collection, analysis, or manuscript preparation.

## References

[1] (2024). 2024 Alzheimer’s disease facts and figures. Alzheimers Dement..

[2] Jack C.R., Bennett D.A., Blennow K., Carrillo M.C., Dunn B., Haeberlein S.B. (2018). NIA-AA research framework: toward a biological definition of Alzheimer’s disease. Alzheimers Dement.

[3] Bateman R.J., Xiong C., Benzinger T.L.S., Fagan A.M., Goate A., Fox N.C. (2012). Clinical and biomarker changes in dominantly inherited Alzheimer’s disease. N Engl J Med.

[4] Baker J.E., Lim Y.Y., Pietrzak R.H., Hassenstab J., Snyder P.J., Masters C.L. (2017). Cognitive impairment and decline in cognitively normal older adults with high amyloid-β: a meta-analysis. Alzheimers Dement (Amst.).

[5] Donohue M.C., Sperling R.A., Salmon D.P., Rentz D.M., Raman R., Thomas R.G., Australian Imaging, Biomarkers, and Lifestyle Flagship Study of Ageing (2014). The preclinical Alzheimer cognitive composite: measuring amyloid-related decline. JAMA Neurol.

[6] Livingston G., Huntley J., Liu K.Y., Costafreda S.G., Selbæk G., Alladi S. (2024). Dementia prevention, intervention, and care: 2024 report of the lancet standing commission. Lancet.

[7] Booth S., Talegawkar S., Fung T., Hoelscher D.M., Anderson C.A.M., Deierlein A. (2024). Dietary patterns and risk of cognitive decline, dementia, Alzheimer’s disease, and mild cognitive impairment: a systematic review. Alexandria (VA): USDA Nutrition Evidence Systematic Review.

[8] Agarwal P., Barnes L.L., Dhana K., Liu X., Zhang Y., Beck T. (2024). Association of MIND diet with cognitive decline among black and white older adults. Alzheimers Dement..

[9] Albadrani H.M., Chauhan P., Ashique S., Babu M.A., Iqbal D., Almutary A.G. (2024). Mechanistic Insights into the potential role of dietary polyphenols and their nanoformulation in the management of Alzheimer’s disease. Biomed Pharmacother.

[10] Gratton G., Weaver S.R., Burley C.V., Low K.A., Maclin E.L., Johns P.W. (2020). Dietary flavanols improve cerebral cortical oxygenation and cognition in healthy adults. Sci Rep.

[11] Nie R.-Z., Luo H.-M., Liu Y.-P., Wang S.-S., Hou Y.-J., Chen C. (2024). Food functional factors in Alzheimer’s disease intervention: current research progress. Nutrients.

[12] Kivipelto M., Mangialasche F., Snyder H.M., Allegri R., Andrieu S., Arai H. (2020). World-wide FINGERS network: a global approach to risk reduction and prevention of dementia. Alzheimers Dement..

[13] Merrill A.H. (2011). Sphingolipid and glycosphingolipid metabolic pathways in the era of sphingolipidomics. Chem Rev.

[14] van Meer G., Voelker D.R., Feigenson G.W. (2008). Membrane lipids: where they are and how they behave. Nat Rev Mol Cell Biol.

[15] van Smeden J., Janssens M., Gooris G.S., Bouwstra J.A. (2014). The important role of stratum corneum lipids for the cutaneous barrier function. Biochim Biophys Acta.

[16] Uchiyama T., Nakano Y., Ueda O., Mori H., Nakashima M., Noda A. (2008). Oral intake of glucosylceramide improves relatively higher level of transepidermal water loss in mice and healthy human subjects. J Health Sci..

[17] Leo T.K., Tan E.S.S., Amini F., Rehman N., Ng E.S.C., Tan C.K. (2022). Effect of Rice (Oryza Sativa L.) Ceramides supplementation on improving skin barrier functions and depigmentation: an open-label prospective study. Nutrients.

[18] Takeda S., Yoneda A., Miyasaka K., Manse Y., Morikawa T., Shimoda H. (2022). Comparative study on epidermal moisturizing effects and hydration mechanisms of rice-derived glucosylceramides and ceramides. Int J Mol Sci.

[19] Higurashi S., Haruta-Ono Y., Urazono H., Kobayashi T., Kadooka Y. (2015). Improvement of skin condition by oral supplementation with sphingomyelin-containing milk phospholipids in a double-blind, placebo-controlled, randomized trial. J Dairy Sci.

[20] Mukai K., Takeuchi M., Ohnishi M., Kudoh M., Imai H. (2022). Characterization of ceramides and glucosylceramides of the satsuma mandarin (Citrus Unshiu) fruit. J Oleo Sci.

[21] Ahn Y., Joo Suh H., Hong K.-B., Choi Y., Lee S.J., Jung E.-J. (2024). Oral intake of milk ceramides improves skin hydration, elasticity, and wrinkles around the eyes: a 12-week, randomized, double-blind, placebo-controlled trial. J Funct Foods.

[22] Maeda-Yamamoto M. (2021). The use of functional agricultural products in the functional food labeling system and its challenges after five years. Food Sci Technol Res.

[23] Yuyama K., Igarashi Y. (2022). Linking glycosphingolipids to Alzheimer’s amyloid-ß: extracellular vesicles and functional plant materials. Glycoconj J.

[24] Yuyama K., Takahashi K., Usuki S., Mikami D., Sun H., Hanamatsu H. (2019). Plant sphingolipids promote extracellular vesicle release and alleviate amyloid-β pathologies in a mouse model of Alzheimer’s disease. Sci Rep.

[25] Yuyama K., Sun H., Mitsutake S., Igarashi Y. (2012). Sphingolipid-modulated exosome secretion promotes clearance of amyloid-β by microglia. J Biol Chem.

[26] Yuyama K., Sun H., Usuki S., Sakai S., Hanamatsu H., Mioka T. (2015). A potential function for neuronal exosomes: sequestering intracerebral amyloid-β peptide. FEBS Lett..

[27] Eguchi K., Mukai K., Yuyama K., Kurimoto N., Tanaka A., Katsuyama T. (2021). Attenuating effects of glucosylceramide extracted from Konjac on amyloid ß accumulation in the human brains. Japanese Pharm Thera..

[28] Nasreddine Z.S., Phillips N.A., Bédirian V., Charbonneau S., Whitehead V., Collin I. (2005). The Montreal cognitive assessment, MoCA: a brief screening tool for mild cognitive impairment. J Am Geriatr Soc.

[29] Dubois B., Feldman H.H., Jacova C., Dekosky S.T., Barberger-Gateau P., Cummings J. (2007). Research criteria for the diagnosis of Alzheimer’s disease: revising the NINCDS-ADRDA criteria. Lancet Neurol..

[30] Wechsler D. (1987).

[31] Wechsler D. (2008).

[32] Salthouse T.A. (1996). The processing-speed theory of adult age differences in cognition. Psychol. Rev..

[33] Adachi K., Ijuin M., Otsuki M., Koike A., Ishiai S. (2018). Examining validity of the standardized verbal paired associates learning test. High Brain Funct Res..

[34] McKhann G.M., Knopman D.S., Chertkow H., Hyman B.T., Jack C.R., Jr; Kawas C.H. (2011). The diagnosis of dementia due to Alzheimer’s disease: recommendations from the national institute on aging-Alzheimer’s association workgroups on diagnostic guidelines for Alzheimer’s disease. Alzheimers Dement..

[35] Nakamura A., Kaneko N., Villemagne V.L., Kato T., Doecke J., Doré V. (2018). High performance plasma amyloid-β biomarkers for Alzheimer’s disease. Nature.

[36] Folstein M.F., Folstein S.E., McHugh P.R. (1975). “Mini-Mental State”. A practical method for grading the cognitive state of patients for the clinician. J Psychiatr Res.

[37] Sperling R.A., Aisen P.S., Beckett L.A., Bennett D.A., Craft S., Fagan A.M. (2011). Toward defining the preclinical stages of Alzheimer’s disease: recommendations from the national institute on aging-Alzheimer’s association workgroups on diagnostic guidelines for Alzheimer’s disease. Alzheimers Dement.

[38] Papp K.V., Rofael H., Veroff A.E., Donohue M.C., Wang S., Randolph C. (2022). Sensitivity of the Preclinical Alzheimer’s Cognitive Composite (PACC), PACC5, and Repeatable Battery for Neuropsychological Status (RBANS) to Amyloid Status in Preclinical Alzheimer’s Disease -Atabecestat Phase 2b/3 EARLY Clinical Trial. J Prev Alzheimers Dis..

[39] Hansson O., Blennow K., Zetterberg H., Dage J. (2023). Blood biomarkers for Alzheimer’s disease in clinical practice and trials. Nat Aging.

[40] Jack C.R., Andrews J.S., Beach T.G., Buracchio T., Dunn B., Graf A. (2024). Revised criteria for diagnosis and staging of Alzheimer’s disease: Alzheimer’s association workgroup. Alzheimers Dement..

[41] Nilsson Å (1969). Metabolism of cerebroside in the intestinal tract of the rat. Biochim Biophys Acta.

[42] Ishikawa J., Takada S., Hashizume K., Takagi Y., Hotta M., Masukawa Y. (2009). Dietary glucosylceramide is absorbed into the lymph and increases levels of epidermal sphingolipids. J Dermatol Sci.

[43] Sugawara T., Tsuduki T., Yano S., Hirose M., Duan J., Aida K. (2010). Intestinal absorption of dietary maize glucosylceramide in lymphatic duct cannulated rats. J Lipid Res.

[44] Kimata H. (2006). Improvement of atopic dermatitis and reduction of skin allergic responses by oral intake of Konjac ceramide. Pediatr. Dermatol..

[45] Eguchi K., Mikami D., Sun H., Tsumita T., Takahashi K., Mukai K. (2020). Blood-brain barrier permeability analysis of plant ceramides. PLoS One.

[46] Usuki S., Tamura N., Yuyama K., Tamura T., Mukai K., Igarashi Y. (2018). Konjac Ceramide (kCer) regulates NGF-induced neurite outgrowth via the Sema3A signaling pathway. J Oleo Sci.

[47] Asanuma N. (2022). Effect of dietary ceramide and glucosylceramide on the alleviation of experimental inflammatory Bowel disease in mice. J Oleo Sci.

